# From genomics and epigenomics to finding hidden regulators

**DOI:** 10.1186/gb4164

**Published:** 2014-02-25

**Authors:** Hailing Jin

**Affiliations:** 1 Department of Plant Pathology and Microbiology, Center for Plant Cell Biology, Institute for Integrative Genome Biology, University of California, Riverside, CA 92521, USA

## Abstract

A report on the Plant and Animal Genome XXII meeting held in San Diego, California, USA, January 11–15, 2014.

## 

The Plant and Animal Genome XXII meeting was held in the warm and sunny coastal city of San Diego, California, on 11–15 January this year, during a time when many other US cities were plagued with snowstorms and extreme cold weather. More than 2,800 attendees from 62 countries joined the meeting. There were eight plenary talks and many workshops and posters covering topics ranging from genomics and functional genomics to epigenomics and systems biology. Tremendous progress in our understanding of the structure, evolution and regulation of plant and animal genomes has been made in recent years. Revolutionary high-throughput sequencing techniques have enabled complete sequencing of many large and complex genomes containing extensive transposable elements (TEs) and repeats, and have also helped in the identification of many hidden regulators, such as small RNAs, especially in non-model species. Here, I highlight some of the exciting presentations on epigenomics and small RNAs.

### Transposons and epigenetic silencing

Whole genome sequencing analysis has revealed that TEs constitute more than half of the genomes of many animals and crop plants. For example, more than 80 % of the maize genome and 60 % of our human genome is composed of TEs. Many studies and presentations support the notion that proliferation of TEs is one of the major driving forces for genome expansion. Nathan Springer (University of Minnesota, USA) described the complex interplay between TEs and genes within the maize genome. He showed that although DNA methylation and a repressive histone methylation marker, H3K9me2, are present in almost all TE regions in maize, DNA methylation patterns and their ability to spread to surrounding regions vary among different transposon families. It was also shown that many genes with TEs in their introns are still actively transcribed, suggesting that maize transcription machinery can tolerate blocks of heterochromatin.

Although TE transposition and replication are beneficial for adaptive evolution in many eukaryotic organisms, uncontrolled transposition activities can be detrimental and even fatal. Therefore, TEs are usually under tight epigenetic control via DNA methylation or histone modifications. In plants, the 24-nucleotide heterochromatic small interfering RNAs (siRNAs) are mainly responsible for silencing TEs by guiding *de novo* DNA methylation and histone modifications. Nathaniel Street (Umea University, Sweden) presented the genome assembly and small RNA profiles of Norway spruce (*Picea abies*), a tree with a similar number of genes as the model plant *Arabidopsis*, but which has a genome 100 times larger and with many more TEs. The large expansion of the genome is due to the slow and steady accumulation of a diverse set of long terminal repeat (LTR) retrotransposons. The 24-nucleotide class of siRNAs is much less abundant in gymnosperms than in angiosperms, which may partially explain the mass proliferation of TEs in gymnosperms. By comparative analysis with related species, it was found that the distribution of small RNA classes was highly diverse and tissue-specific. In animals, PIWI proteins and PIWI-interacting small RNAs (piRNAs) are key regulators in suppressing TE proliferation in germ cells. Michael Vandewege (Mississippi State University, USA) discussed the interplay between piRNAs and TEs among Laurasiatherian mammals. Small RNAs from the testes of dog, cat, vesper bat and horse were profiled and it was shown that piRNAs play an essential role in suppressing the expression of active retrotransposons in mature testes in dog, cat and horse, whereas the DNA transposons in the vesper bat, which lacks a RNA intermediate, are not targeted by piRNAs. This demonstrates the complex dynamics between TEs and genomic defenses in mammalian genomes.

Despite robust genome integrity maintenance, certain TEs can still circumvent the epigenetic surveillance and amplify rapidly in the genome without killing its host or being silenced. Sue Wessler (University of California, Riverside, USA) gave a plenary talk on one such successful TE - a miniature inverted repeat transposable element (MITE), *mPing*, in rice. In a few rice landraces, *mPing* has been amplified to more than 750 copies during recent domestication. She further illustrated that one strategy for successful rapid amplification is that *mPing* has a preference for inserting into AT-rich regions and thus minimizes exon insertions because rice protein coding regions tend to be GC-rich. This study has provided an excellent example that links TEs with genome restructuring and regulation.

Caroline Dean (John Innes Centre, UK) gave a plenary talk on co-transcriptional coupling mechanisms that link chromatin dynamics with antisense transcripts for quantitative gene regulation. At the flowering repressor *FLOWERING LOCUS C* (*FLC*) locus, the antisense transcripts COOLAIR are alternatively spliced and polyadenylated, which recruit distinct chromatin-modifying complexes for different *FLC* chromatin states. *FLC* is one of the best-studied gene loci that are under precise control via multiple layers of regulation at the epigenetic level.

The plant and animal epigenome workshops were held separately during the conference. Rob Martienssen (Cold Spring Harbor Laboratory, USA) and Eric Lyons (University of Arizona, USA) introduced the Epigenomics of Plants International Consortium (EPIC), a joint effort to support the community working on plant epigenomics and epigenetics. In plants, epigenetic control is associated with the RNA-directed DNA methylation (RdDM) pathway. Plant-specific RNA polymerase IV (Pol IV) is a central player that initiates siRNA biogenesis in the RdDM pathway. Julie Law (Salk Institute for Biological Studies, USA) presented work on the characterization of SAWADEE HOMEODOMAIN HOMOLOG 1 (SHH1), a protein that targets Pol IV to chromatin. She found that SHH1 was able to recognize both H3K9 methylation and an unmodified H3K4 residue, which connects DNA methylation and H3K9 methylation within the context of the RdDM pathway. Blake Meyers (University of Delaware, USA) discussed phased secondary siRNAs (phasiRNAs) in plants. He compared phasiRNAs and their generation loci between dicots (*Arabidopsis*, soybean and *Medicago truncatula*) and monocots (*Brachypodium distachyon*, rice and maize), and discovered a novel class of 24-nucleotide phasiRNAs from monocots that are generated by a monocot-specific Dicer-like (DCL) protein. This class of phasiRNAs, emerging with germinal cell maturation and meiosis, shares characteristic features with animal piRNAs. Doris Wagner (University of Pennsylvania, USA) introduced the function of ATP-dependent SWI/SNF chromatin remodeling proteins, SPLAYED and BRAHMA, during lateral organ formation in *Arabidopsis*. Meanwhile, the animal epigenetics workshop covered topics ranging from genomic imprinting and transcriptome analysis to public resources, such as the livestock epigenetics database (epiDB).

### Small RNAs

Small RNAs are short non-coding regulatory RNAs that direct gene silencing in a sequence-dependent manner. It has been 20 years since the first microRNA (miRNA) was discovered in *Caenorhabditis elegans*. Since then, small RNA-mediated gene silencing has set a new paradigm for understanding eukaryotic gene regulation. Pamela Green and Blake Meyers (University of Delaware, USA) have organized informative ‘Small RNA’ workshops at this meeting for the last eight years. This year, six speakers from both animal and plant fields presented their findings. In addition, small RNAs have become an indispensable part of genomics studies and appeared in many genomics and epigenomics presentations.

Several talks addressed the function of small RNAs in various cellular processes. For example, Alexandre Colas (University of California, San Diego, USA) presented findings on the function of several miRNAs, including *let-7*, in cardiovascular differentiation using whole genome miRNA screening, revealing a remarkable conservation of function from amphibians to mammals. Robert Sullivan (Chu de Quebec Research Center, Canada) reported findings on extracellular microvesicle-associated miRNAs in the male reproductive tract. Distinct miRNA repertories were found in the epididymal intraluminal compartment that are likely to be involved in intercellular communication through the epididymis via extracellular microvesicles. Beth Thompson (East Carolina University, USA) introduced the function of DCL1 in maize development. Pamela Green gave a talk on the genome-wide analysis of *Brachypodium* miRNAs in the Brachypodium Genomics workshop. Conserved and novel miRNAs were identified and their potential targets were predicted and confirmed by parallel analysis of RNA ends.

In addition to their role in animal and plant development, small RNAs also play essential roles in regulating host-pathogen interactions. I (Hailing Jin, University of California, Riverside, USA) presented our work on pathogen small RNAs that regulate host immunity in the ‘Small RNAs’ and ‘Host-Microbe Interactions’ workshops. A group of small RNAs from an aggressive fungal pathogen, *Botrytis cinerea*, can be delivered into host plant cells to silence host immunity genes by hijacking host RNA interference (RNAi) machinery to facilitate its infection. This finding represents a naturally occurring cross-kingdom RNAi, which serves as an advanced virulence mechanism for aggressive eukaryotic pathogens. Barbara Baker (University of California, Berkeley, USA) described analyses of several plant endogenous small RNAs that regulate host immunity. The tobacco *N* gene that confers resistance to tobacco mosaic virus is regulated by both promoter-targeted transposon MITE-associated siRNAs and coding-region-targeted miRNAs. They also identified a new class of miRNAs derived from long inverted repeats, one of which targets *Cf9*, a tomato gene that confers resistance to fungal pathogen *Cladosporium fulvum*.

Brian Gregory (University of Pennsylvania, USA) showed that the specific secondary structure of mRNAs can act as a potent *cis*-regulatory feature through small RNA processing machinery in *Arabidopsis*. By performing RNA-seq on double-stranded RNA and single-stranded RNA pools, a negative correlation between mRNA abundance and structure complexity was identified. Thus, in addition to long double-stranded RNAs or miRNA precursors, highly structured mRNAs can also potentially serve as substrates of DCL proteins and give rise to small RNAs with a wide size range, unveiling a new regulatory function of DCLs on highly structured mRNAs.

### Conclusions

As the premier and largest ag-genomics meeting in the world, this year’s Plant and Animal Genome Conference was a great success, and there are many more exciting and stimulating talks that I unfortunately do not have space to discuss here. Even if you were overwhelmed by the wealth of information presented at the meeting, you would have certainly been inspired. It was very satisfying to see that epigenetics and small RNAs have become an important part of many genomics studies, especially in complex non-model systems, which indicates their essential roles in regulating genes and genomes.

### Abbreviations

DCL: Dicer-like; miRNA: microRNA; MITE: Miniature inverted repeat transposable element; phasiRNA: phased secondary siRNA; piRNA: PIWI-interacting small RNA; Pol IV: Polymerase IV; RdDM: RNA-directed DNA methylation; RNAi: RNA interference; SHH1: SAWADEE HOMEODOMAIN HOMOLOG 1; siRNA: small interfering RNA; TE: Transposable element.

### Competing interests

The author declares that she has no competing interests.

